# Research provides evidence for health policy in Lao PDR

**DOI:** 10.1080/16549716.2020.1791415

**Published:** 2020-08-03

**Authors:** Ian Bromage, E. Pamela Wright, Sengchanh Kounnavong, Vanphanom Sychareun, Léonie Venroij

**Affiliations:** a Consultant for MCNV, Hanoi, Vientiane; b Guelph International Health Consulting: Amsterdam, The Netherlands; c Director, Lao Tropical and Public Health Institute, Ministry of Health, Vientiane Capital, Lao PDR; d Dean, Faculty of Public Health, University of Health Sciences, Vientiane Capital, Lao PDR; e LEARN Program Manager, MCNV Laos, Vientiane Capital, Lao PDR

## Introduction

Recent decades have seen the growth of a requirement for evidence to inform the development of health policies around the world. In 2015, the European Union initiated a programme to strengthen the capacity of national institutes of public health in several low- and middle-income countries to provide such evidence.

In this Special Issue of *Global Health Action*, papers are presented that address a number of research questions aimed at providing the Lao Ministry of Health with evidence on topics that had high priority for them, mainly relating to reproductive health and nutrition.

The Lao People’s Democratic Republic (Lao PDR) faces a range of sexual and reproductive health (SRH) challenges, including poor sexual and reproductive health (SRH) literacy, low uptake and use of contraceptives, and the resulting unintended pregnancies. These pregnancies lead to school dropout and early marriage, and one of the highest abortion rates in South-east Asia. Child malnutrition, inadequate coverage of national health insurance and inaccessible, low-quality services further aggravate these problems. The research reported in the papers in this special issue was conducted within the framework of a much larger initiative supported by the European Union (EU): the Supporting Public Health Institutes Program (SPHIP).

## SPHIP and the LEARN programme in Lao PDR

The goal of the EU programme SPHIP is to strengthen national public health institutes (NPHIs) in eight low-income countries, including Lao PDR. The aim is to enable them to provide national authorities with evidence-based and locally relevant advice to support decision-making and health policy development, and to contribute to monitoring and evaluating the effectiveness of policies and programmes [1].

In Lao PDR, the SPHIP initiative focuses on strengthening the capacities of Lao Tropical and Public Health Institute (Lao TPHI) and the University of Health Sciences (UHS). The multi-year project is called **L**ao **E**quity through policy **A**nalysis and **R**esearch **N**etworks (LEARN). The name reflects the importance of Lao public health research in supporting the Government of Lao PDR (GoL) to meet its targets for Universal Health Coverage and to achieve other health-related Sustainable Development Goals [2]. It also reflects the importance of continuous learning through high quality research.

The LEARN Programme fosters south-south co-operation with the Hanoi University of Public Health (HUPH) in Vietnam, with additional north-south support from the Vrije University (VU), the Netherlands. The collaborative management model is coordinated by the Vientiane office of the Dutch international NGO, MCNV, which has worked in health development in Vietnam and Lao PDR for decades. The institutes and MCNV have been working closely with the EU Delegation in Lao PDR to implement the programme according to the objectives of the EU programme and local aspirations.

## Functions, attributes and constraints of public health institutes in Lao PDR

The International Association of NPHIs defines a national public health institute as *“an organization, or group of organizations, that provides critical resources to the Government and other public health bodies for guiding policy-making, establishing public health priorities, evaluating the implementation of public health policies and programmes* [3]. In Lao PDR, two key institutes engaged in public health are Lao TPHI and UHS. With a focus on the major health issues affecting Lao PDR and as trusted counsels to the Government, they have a national scope of influence and legitimacy. Together with other organisations, such as medical service providers and national laboratories, these two institutes play a crucial role in assisting the GoL to design and deliver long-term, sustainable improvements to the health of the population.

Lao TPHI is responsible for analysis and evaluation of population health, health and disease surveillance, monitoring of emerging threats to public health, and promotion of coverage and access to health services. The UHS has the main role in human resource development and training. Both institutes had the ambition to strengthen their capacity and increase their resources, especially to undertake high quality public health research to generate reliable data that are collected, analysed and interpreted to international standards. The research results must be translated into clear evidence for policy makers, and provide the information needed to engage communities in disease prevention and health promotion.

To fulfil their roles, Lao TPHI and UHS need adequate human and financial resources and essential infrastructure, including reliable internet connections and access to scientific literature. They also want to co-ordinate their activities with other organizations, not only within Lao PDR but also in regional and global networks, to find solutions to shared health problems.

However, in Lao PDR, as in many LMICs, public health research has been underfunded in the face of more immediate health pressures. Further, public health interventions may take a longer time than do clinical interventions to show improved health outcomes, which tends to push government funding towards hospitals and clinics. Developing the capacity of public health institutes in these contexts requires long-term commitment and often, external support.

## Addressing the constraints through the LEARN Programme

The overall goal of the LEARN programme is to contribute to improved population health and the attainment of universal health care in Lao PDR through better-informed health policies and monitoring and evaluation of their implementation. LEARN has two specific objectives: firstly, to support Lao public health institutes to strengthen their capacity to provide national health authorities and other stakeholders with evidence, and secondly, to support Lao TPHI and UHS to provide evidence from high quality health research to the GoL, with particular focus on two key priorities, reproductive health and nutrition.

The LEARN programme was based upon the requirements of the institutes at the time that it was designed. The programme took a holistic approach to enhancing their capacity over a five-year period (2015–2020). Investments were based on needs identified by Lao TPHI and UHS. One area of intervention was IT infrastructure, which was upgraded so that both institutes had improved internet access and connectivity. Lao TPHI improved the functionality of its website and the on-line research portal, which provides public access to a database of research undertaken within Lao PDR. An electronic library system was introduced at UHS along with additional learning materials enabling students and researchers to access international academic journals and databases. Conferencing facilities were improved; meeting spaces and study areas were made brighter, more attractive spaces; a minibus was purchased to take students to the field for learning and to take researchers to study sites. These investments in physical infrastructure were crucial for an improved research environment. However, the most significant activities of LEARN were focused upon developing human resources and training the next generation of researchers, who could design and implement studies relevant to the public health priorities of Lao PDR. The result would be more and better quality evidence for informing policy makers and monitoring policy implementation.

## Enhancing the quality and value of public health research in Lao PDR

To facilitate selection of research topics closely related to health care priorities in Lao PDR, LEARN supported the formulation of a National Health Research Agenda (NHRA) [4]. The approach to generating this agenda was to combine results from an assessment of the current health policy and implementation gaps, in-depth interviews with stakeholders, and a survey of health service providers at district, provincial and national levels. A set of health themes clearly emerged, which were ranked by a panel of Lao public health experts, using the Delphi technique, to set health research priorities. The themes were further sub-divided into focused topics, incorporated into the published NRHA. A more detailed description of the process to formulate the agenda, by Essink et al. [5], is provided in this issue.

A key focus of the LEARN programme has been the development of human resources within, and available to, public health institutes. LEARN brought the UHS together with the Hanoi University of Public Health to develop the first international Master in Public Health (MPH) Programme [6] to be run in Lao PDR. The MPH curriculum is tailored to the requirements of the NRHA. The Lao students undertake their studies at UHS and HUPH and conduct their research in Lao PDR. The UHS has also made use of technical assistance from the Hanoi partner to improve the quality of their own teaching including their MPH training. In short courses, Lao TPHI has been improving their research training both for researchers and for the Ethics Review Committee; the training serves not only their own staff but researchers from other institutes including the UHS. These developments have contributed to better teaching on research and public health and improved training for researchers already working in the institutes.

In addition to the MPH, the LEARN programme supports five PhD students who are undertaking their research as part of VU’s Global Public Health Programme. These PhD students work together with both Lao and Dutch MPH students and are supported by senior Lao researchers who are also conducting post-doctoral studies. The studies recognize the complexities of public health issues and emphasize transdisciplinary approaches, for example, the SRHR studies examine gender, cultural and educational perspectives.

Throughout the LEARN programme, short courses are provided by national and international experts in topics such as qualitative research methods, transdisciplinary approaches, data collection and analysis, presentation skills, and producing policy briefs. To ensure the research is conducted to the highest ethical standards and guarantee the safety of research participants, specific training and support was provided to strengthen the Lao National Ethics Review Committee and the establishment of documented procedures for the review and approval of research undertaken in Lao PDR.

### The papers in this Special Issue

The fourteen research papers in this Special Issue represent a selection of research undertaken within the LEARN framework; more than a dozen papers have been published in international journals during previous years. The papers cover the LEARN priority areas of sexual and reproductive health, nutrition and universal health coverage.

The first paper describes the capacity building action around the development of the National Health Research Agenda [5]. The agenda is currently embedded within MoH, and aids in selecting research topics.

The nine papers in this issue that address SRH focus predominantly on the emerging need for information about adolescent SRH. Laos has a large adolescent population and a high rate of teenage pregnancy. At the onset of LEARN, knowledge on these issues was lacking, but needed for policies and interventions. A series of papers address adolescent sexual and reproductive health from different points of view. Vongxay et al. investigated adolescent attitudes to abortion [7], Phongluxa et al. considered sexual knowledge and practice of adolescents [8], Sychareun et al. menstruation management among adolescents [9], Santisouk et al. pregnancy literacy [10], Vongsavanh et al. discussions about sex between adolescents and their parents [11], and Inthavong et al. safe sex and sexually transmitted infections [12]. Insights from these studies are currently being applied in policy and planning for sexual education in and out of schools and the scale-up of youth friendly RH services. Unmet need of family planning remains also a critical issue in Lao, particularly but not only among adolescents. Chanthakoummane et al. [13] investigated contraceptive use from the perspective of gender roles while Thongmixay et al. [14] assessed the quality of family planning services available to women of reproductive age. In a paper about how health service staff deal with stillbirth, Choumannivong et al. [15] make the first steps in developing guidelines to address this sensitive issue.

Kounnavong et al. [16] bridged the topics of adolescence and nutrition with their study revealing the high rates of anaemia among high-school students. The paper by Boulom et al. [17] demonstrates that ethnic minority children in remote mountainous areas are greatly affected by malnutrition. For example, stunting levels were 72%, more than twice the national average. This study is particularly relevant for national policy and planning, as Lao PDR has many vulnerable, hard-to-reach areas that are not benefitting equally from successes in addressing malnutrition on a national level.

In the process of reaching universal health coverage, the MoH has rolled out the national health insurance scheme. LEARN contributed an evaluation of the scheme, focused on whether the benefits are reaching Lao citizens, especially the vulnerable. Chaleunvong and colleagues provided two articles revealing that implementation is only partly effective [18], with about 20% of subscribers paying above the defined amount of (co-)payment for out- and in-patient services, and most citizens were not aware which scheme they were enrolled in and what their entitlements were [19]. The authors are working with MoH to address these issues.

## Conclusion: translating knowledge into policy and action

The LEARN programme aimed to contribute to improved health outcomes in Lao PDR through better informed health policies based on evidence from strong research. Knowledge generated through research needs to be translated into policy and actions [20] so to guide this process, LEARN supported the development of a Knowledge Translation Strategy (KTS) [21]. The KTS is meant to ensure the greater involvement of key stakeholders, such as the MoH, in the research process and to make research more accessible to different audiences, with the goal of informing health policies and practices through the use of clear, objective evidence.

Because several research projects aimed to gain information about youth and reproductive health, the Lao Youth Union (LYU) was also invited to participate in and to organize activities around both collection and dissemination of research results. For example, findings on health literacy among adolescents and on teenage pregnancy were shared in a research café with LYU as well as ministries, services, research institutes and international partners. This session concluded by identifying activities to increase health literacy and reduce teenage pregnancy, based on research findings. A follow-up activity was a video contest managed by LYU, asking adolescents to submit videos expressing their perspectives on contraception and pregnancy. In preparing their strategic plan 2020–2025, the LYU included the information from these sessions and invited the researchers (two of the LEARN PhD candidates) to review and contribute to the plan.

Part of the KTS supports attendance at international conferences to facilitate linking and learning among partners in the EU Supporting Public Health Institutes Programme, and to foster more and stronger North-South linkages and South-South collaborations. At a regional level, the LEARN programme has supported the Greater Mekong Sub-region Public Health Conference, which brings together public health institutes and universities from Thailand, Cambodia, Myanmar, China, Vietnam and Lao PDR. It has also supported the annual National Health Research Forum and an annual Adolescent Health Research Day in Lao PDR in cooperation with the country office of UNFPA. All of these provide opportunities for showcasing Lao health research, for engaging with peers from around the world, and for informative dialogues with political leaders and other key stakeholders, such as the WHO.

As well as supporting formal academic research conferences, the LEARN programme helped to establish more informal settings where research could be discussed, such as a research ‘café’ where researchers could share their experiences and support each other. IT facilitated the translation of research findings and recommendations into concise policy briefs accessible to busy decision-makers. Research was presented using different formats, such as film and video, and disseminated via social media, such as Facebook, to inform and engage the public in the research process. Finally, the LEARN programme also supported the publication of Lao research papers in peer reviewed international journals such as *Global Health Action*.

All of these processes combined will help to ensure that Lao public health policies are underpinned by clear evidence, adapted and suitable to the Lao context, and contribute to more effective implementation, to the benefit of all Lao people.

This introduction to the LEARN special issue has been prepared by people who were closely involved in the program from the early stages: Mr. Ian Bromage, former Country Director, MCNV Lao PDR, Dr. E. Pamela Wright, former General Director, MCNV, Dr. Vanphanom Sychareun, Dean, Faculty of Public Health, University of Health Sciences, Dr. Sengchanh Kounnavong, Director, Lao Tropical and Public Health Institute, Ms. Léonie Venroij, LEARN Programme Manager, MCNV Lao PDR.
